# Factors affecting the transmission of SARS‐CoV‐2 in school settings

**DOI:** 10.1111/irv.12968

**Published:** 2022-02-10

**Authors:** Haokun Yuan, Connor Reynolds, Sydney Ng, Wan Yang

**Affiliations:** ^1^ Department of Epidemiology, Mailman School of Public Health Columbia University New York NY USA; ^2^ Department of Biostatistics, Mailman School of Public Health Columbia University New York NY USA

**Keywords:** children, COVID‐19, preventive measures, school, secondary attack rate

## Abstract

**Background:**

Several studies have reported SARS‐CoV‐2 outbreaks in schools, with a wide range of secondary attack rate (SAR; range: 0–100%). We aimed to examine key risk factors to better understand SARS‐CoV‐2 transmission in schools.

**Methods:**

We collected records of 35 SARS‐CoV‐2 school outbreaks globally published from January 2020 to July 2021 and compiled information on hypothesized risk factors. We utilized the directed acyclic graph (DAG) to conceptualize risk mechanisms, used logistic regression to examine each risk‐factor group, and further built multirisk models.

**Results:**

The best‐fit model showed that the intensity of community transmission (adjusted odds ratio [aOR]: 1.11, 95% CI: 1.06–1.16, for each increase of 1 case per 10 000 persons per week) and individualism (aOR: 2.72, 95% CI: 1.50–4.95, above vs. below the mean) was associated higher risk, whereas preventive measures (aOR: 0.25, 95% CI: 0.19–0.32, distancing and masking vs. none) and higher population immunity (aOR: 0.57, 95% CI: 0.46–0.71) were associated with lower risk of SARS‐CoV‐2 transmission in schools. Compared with students in high schools, the aOR was 0.47 (95% CI: 0.23–0.95) for students in preschools and 0.90 (95% CI: 0.76–1.08) for students in primary schools.

**Conclusions:**

Preventive measures in schools (e.g., social distancing and mask wearing) and communal efforts to lower transmission and increase vaccination uptake (i.e., vaccine‐induced population immunity) in the community should be taken to collectively reduce transmission and protect children in schools.

## 1 INTRODUCTION

Since the early stages of the COVID‐19 pandemic, concerns have been raised about the impact of schools on community transmission and the well‐being of students and staff, as well as the impact on the schedules of healthcare workers concerning childcare.^1^ Out of an abundance of caution and fear that the SARS‐CoV‐2 virus would spread rapidly in schools much like influenza pandemics,^2^ countries globally decided to suspend in‐person classes and begin online instruction. By April 2020, over 600 million students worldwide were affected by school closures in response to the COVID‐19 pandemic.^3^ In contrast to influenza pandemics where children are the key drivers of transmission, studies have indicated that children are likely less susceptible to SARS‐CoV‐2 infection, tend to experience less severe disease when infected, and likely have lower transmissibility.^4^, ^5^ Given this new evidence, schools in many places have gradually reopened since the summer of 2020, while implementing varying level of preventive measures (e.g., mask wearing, distancing, limiting the number of students, rotating schedules, and viral testing) to reduce risk of transmission. Given these circumstances, the risk of SARS‐CoV‐2 outbreaks in school settings may differ substantially across space and time. Indeed, several studies have examined school outbreaks of COVID‐19 and reported secondary attack rates (SARs)—that is, the proportion of infected contacts of an index case out of all contacts of that index case^6^—among students ranging from 0% (i.e., no secondary infections) to 100% (i.e., infections among all contacts). However, this discrepancy is still not fully understood, and a better understanding can inform better preventive measures for future outbreaks not limited to COVID‐19 or school settings.

To identify the main factors that determine the transmission of SARS‐CoV‐2 in schools and inform strategies to prevent future school outbreaks, here, we examined the associations between SARS‐CoV‐2 SAR in children and various potential risk factors. We compiled data from relevant studies in the literature reporting SARS‐CoV‐2 SAR in schools and for related factors (e.g., incidence in the community and population immunity cumulated over time) and further used regression models to examine key risk factors of having high SAR in schools. Consistent with previous work, we found the risk varied by school level, with lower risk among preschool and primary school students than high schoolers. Accounting for school level, we found that implementation of preventive measures (distancing and mask wearing) in schools and higher population immunity were associated lower SAR in schools; in contrast, higher SARS‐CoV‐2 transmission in the community and higher level of individualism were associated with higher SAR in schools.

## 2 METHODS

### 2.1 Data sources

**FIGURE 1:**
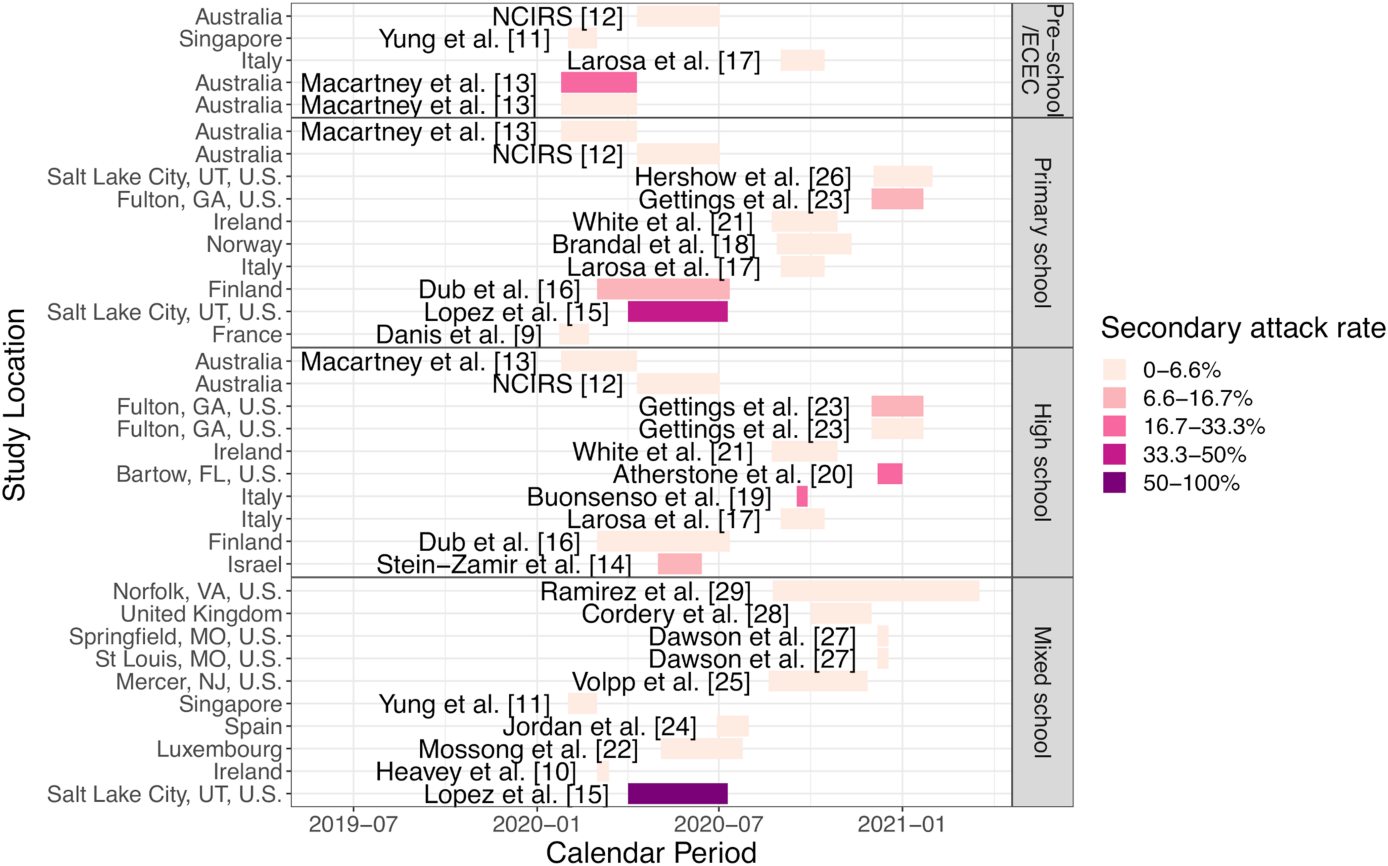
SARS‐CoV‐2 school outbreak studies included in the analysis.^9^, ^10^, ^11^, ^12^, ^13^, ^14^, ^15^, ^16^, ^17^, ^18^, ^19^, ^20^, ^21^, ^22^, ^23^, ^24^, ^25^, ^26^, ^27^, ^28^, ^29^ Each colored bar represents an observed school outbreak; the school location is shown on the *y*‐axis, and study period is shown by the position and length of the bar (see calendar time on the *x*‐axis); school type is shown in the panel title on the right; and reported secondary attack rate (SAR) is indicated by the color of the bar (see the legend)

Studies were searched for on the “Living Evidence for COVID‐19” database,^7^ which retrieves articles from EMBASE via Ovid, PubMed, BioRxiv, and MedRxiv. Any article within this database was considered, from December 2019 up to July 28, 2021. The search terms used include “transmission AND (school OR schools)” or “transmission AND children.” A total of 727 articles were found using these search terms. When titles and abstracts were identified as being potentially relevant, the articles were read to determine if an outbreak (defined as at least one case reported) took place in a school setting and if the number of infections and contacts among students were reported. That is, here, we restricted our analyses to school outbreaks and secondary infections among students. In addition, we extracted 11 observations included in a systematic review of evidence regarding the ability of children to transmit SARS‐CoV‐2 in schools.^8^ In total, 35 school outbreaks extracted from 21 articles were included in this analysis (see Figure 1 and Supporting Information).

Relevant data, as deemed by an initial conceptual analysis using the directed acyclic graph (DAG; see details below), were taken from the articles identified above. These included the time period of the study, study design, location, age of children, type of school according to the International Standard Classification of Education,^30^ reported SARs among students, number of contacts of the index case, testing method (PCR vs. serology), level of surveillance (all contacts, some contacts, only symptomatic), and whether masks and social distancing were required. In addition, we compiled additional data for potential risk or confounding factors of SARS‐CoV‐2 transmission in schools for each identified study as detailed in the next section.

### 2.2 Conceptual analysis and variable coding

**FIGURE 2:**
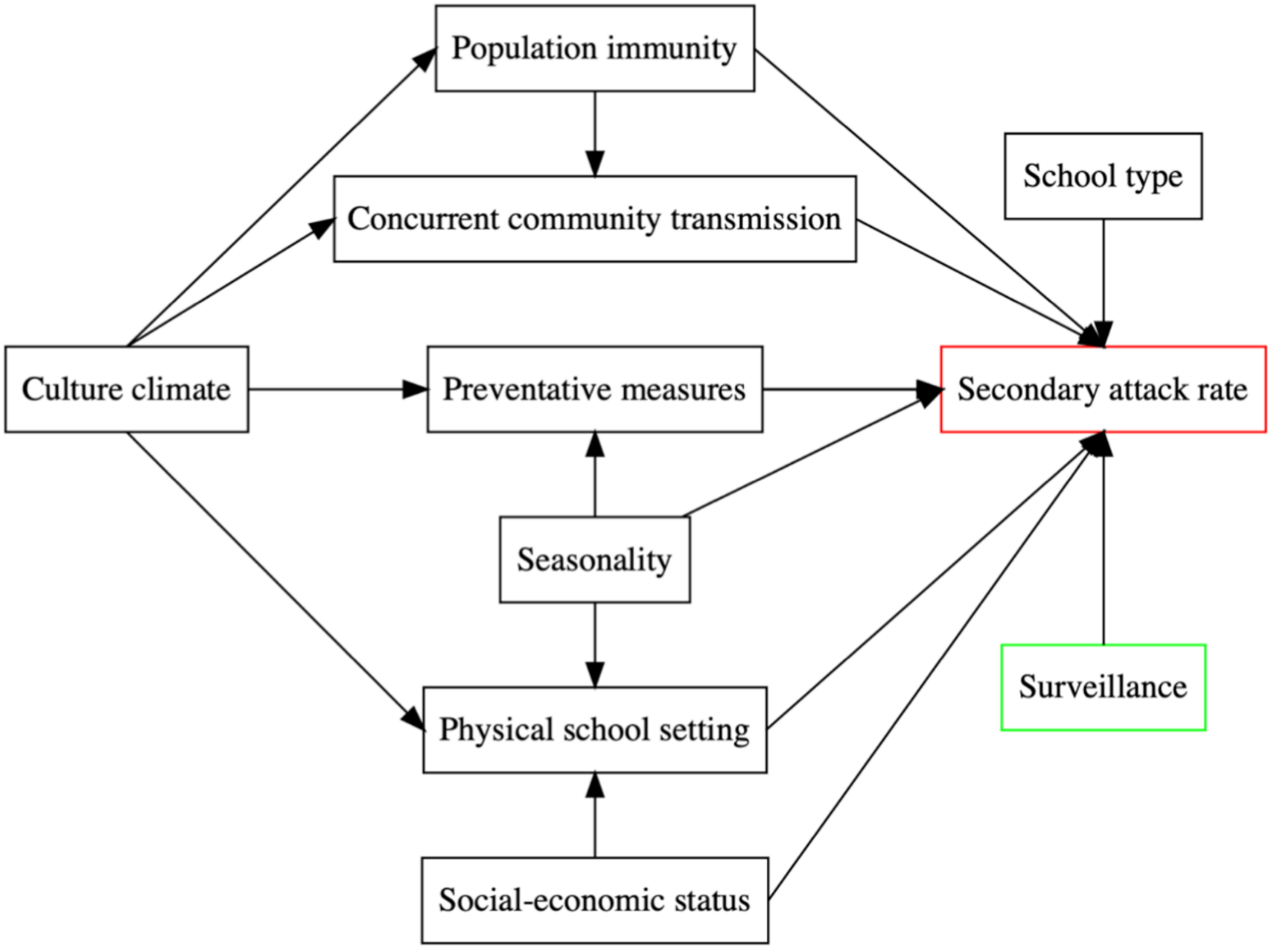
Directed acyclic graph (DAG) describing the relationship among variables. This DAG represents the meaningful relationships between the variables relevant to SARS‐CoV‐2 SAR among children in school settings and informs all further analyses. The outcome measurement, SAR, is presented in red, whereas risk factors are in black and surveillance in green

The unit of analysis was individual outbreak, and in cases where several school types were covered in one study, the data were stratified by those school types. We first conducted a conceptual analysis using the DAG and identified nine key components that may affect SARS‐CoV‐2 transmission in schools (Figure 2). Below, we describe each of the nine components, rationale for inclusion, and related variables examined.

1. School types, based on studies indicating differential transmission risk among different age groups.^31^, ^32^ Here, we examined this factor as a categorical variable including four levels, that is, preschool or early childhood education center (ECEC), primary school, high school, and mixed‐level school. The first three levels were per reports in the included school studies. For studies that examined several types of school but did not report school type specific SARs, we assigned them to a “mixed‐level school” category. For example, if a study gave the overall SAR combining a preschool and a primary school, it was given the value “mixed‐level school.” SARS‐CoV‐2 SAR among children in school settings is the number of infected contacts divided by the total number of contacts of the index cases at each school.
2. Physical school settings such as student density in the classroom and ventilation systems that may affect the intensity of school contact and clearance of air. As it is difficult to obtain information related to ventilation settings, here, we included class size in our analysis based on the average number of students per classroom in each country, as reported by the Organisation for Economic Co‐operation and Development (OECD).^33^
3. Preventive measures, which may reduce outbreak risk. Here, we categorized this variable based on the implementation of mask wearing and/or social distancing in schools, that is, “No preventive measures” if neither measure was required, “Single preventive measure” if only one measure (i.e., distancing or masking) was required, and “Combined preventive measure” if both were required. Note that we were not able to test distancing and masking separately due to the small sample size of schools that required masking alone (n = 2).
4. Surveillance and/or testing policies implemented in schools. On the one hand, testing policies could affect the reported values of SAR; for instance, testing of all contacts regardless of symptoms may lead to identification of more infections including those asymptomatic and increase the numerator of SAR. On the other hand, frequent testing of all if combined with school closure may serve as a containment measure to reduce the risk of onward transmission and, in turn, reduce SAR. Here, we thus included the reported testing practices for contacts in the school outbreak clusters as a categorical ordinal variable. Three types of testing were reported in the school studies, including testing only the symptomatic, both symptomatic and some asymptomatic, and all contacts. However, due to the small sample size in “only symptomatic” (n = 3), we dichotomized surveillance to testing “only symptomatic or some asymptomatic” and “all contacts” of an index case in each school cluster.
5. Seasonal changes such as humidity and temperature. Such seasonal changes may affect the survival and transmission of SARS‐CoV‐2 as well as human behavior. For the latter, for instance, mask wearing may be less strictly adhered to during hot summer days due to discomfort and, in turn, indirectly affect SAR through the use of preventive measures (Figure 2). These seasonal weather conditions can also affect physical school settings (e.g., classroom air ventilation and allowed class size given air quality). Here, we used specific humidity (a measure of absolute humidity) to examine the potential impact from disease seasonality, as specific humidity and temperature are highly correlated. Specifically, ground surface temperature and relative humidity for each study location were extracted from the National Oceanic and Atmospheric Administration using the “rnoaa” package.^34^ Daily mean specific humidity in g H_2_0/kg air was then computed based on the meteorological data using formula introduced by Bolton^35^ and further averaged over the corresponding study period.
6. Intensity of community transmission. Intense community transmission may increase the introduction of infections into schools. In addition, due to the tight connection between school children and their households and community, it could be challenging to ascertain the source of infection, particularly amid a concurrent community outbreak, which, in turn, could affect the reported values of SAR. To examine this impact, we included two measures, that is, the weekly COVID‐19 case rate and weekly COVID‐19‐related death rate for the study area using data from the John Hopkins Coronavirus Resource Center^36^ and standardized by the corresponding population size (for non‐U.S. sites, country‐level data were used, and for U.S. sites, county‐level data were used). To account for the potential lower detection rate during the early phase of the pandemic (Figure 1) and time lag from infection to death, we extended the time period by 2 weeks when computing community case rates and death rates. However, we also tested models using these measures without the 2‐week extension (see the “Sensitivity analysis” section below).
7. Prior population immunity in the community. Population immunity gained from prior infections or COVID‐19 vaccination could lower population susceptibility and hence the risk of SARS‐CoV‐2 in the community. As most school outbreaks included here occurred prior to the rollout of mass‐vaccination, population immunity at those times would mostly come from natural infections (see Figure 1 for the timeline of each study, vs. earliest vaccination rollout for the general population round spring 2021). Thus, here we used the cumulative COVID‐19 case rate (up to the mid‐point of the corresponding study period) as a proxy to account for prior population immunity.
8. Cultural climates, which “represent independent preferences for one state of affairs over another that distinguish countries (rather than individuals) from each other”^37^ and may reflect the collective risk tendency of a population. The Hofstede's cultural dimensions theory^37^ included six related measures including individualism, masculinity, uncertainty avoidance, long term orientation, and indulgence. In particular, individualism is defined as the degree of interdependence of society maintains among its members. We reasoned that the individualism measure would be most relevant to the level of compliancy to public health interventions and, in turn, the risk of SARS‐CoV‐2 transmission. Thus, here, we included individualism in our analysis and dichotomized the reported values for each country.^38^ Among all study sites included here, the mean of individualism scores was 77; thus, we coded those with a score >77 as “Higher individualism” those with a score ≤77 as “Lower individualism.”
9. Indicators of socioeconomic status such as national income that reflect a country's ability to mobilize resources to fight COVID‐19. As such, we included measured national income for each study in our analysis; specifically, national income is measured as the gross domestic product (GDP) subtracting capital depreciation and adding net foreign income, using data from the World Inequality Database.^39^

### 2.3 Statistical analyses

#### 2.3.1 Marginal analysis

Due to the low number of observations (n = 35 outbreaks), we conducted an initial analysis to test combinations of the DAG covariates described above. The goal was to examine the relationship between the SAR and only one group of variables at a time and then include the most relevant predictors into the final model based on this analysis. For each test, we used a logistic regression model of the following form:

l𝑜𝑔𝑖𝑡(𝑆𝐴𝑅)∼𝑋,

where logit is the log‐odds (i.e., $\log\left(p/1-p\right)$, with *p* as the probability of event) and SAR represents the SAR as reported from each of the 35 outbreaks. *X* is one of the combinations of variables we examined as follows:

1. School type
2. Classroom size, adjusting for national income, seasonal changes, and cultural climate
3. Preventive measures, adjusting for seasonal changes and cultural climate
4. Seasonal changes
5. Community transmission (weekly death rate per 100,000 or weekly case rate per 10,000; i.e., only one measure is included, because these two measures are highly correlated), adjusting for cultural climate, and population immunity
6. Prior population immunity, adjusting for cultural climate
7. National income

**FIGURE 3:**
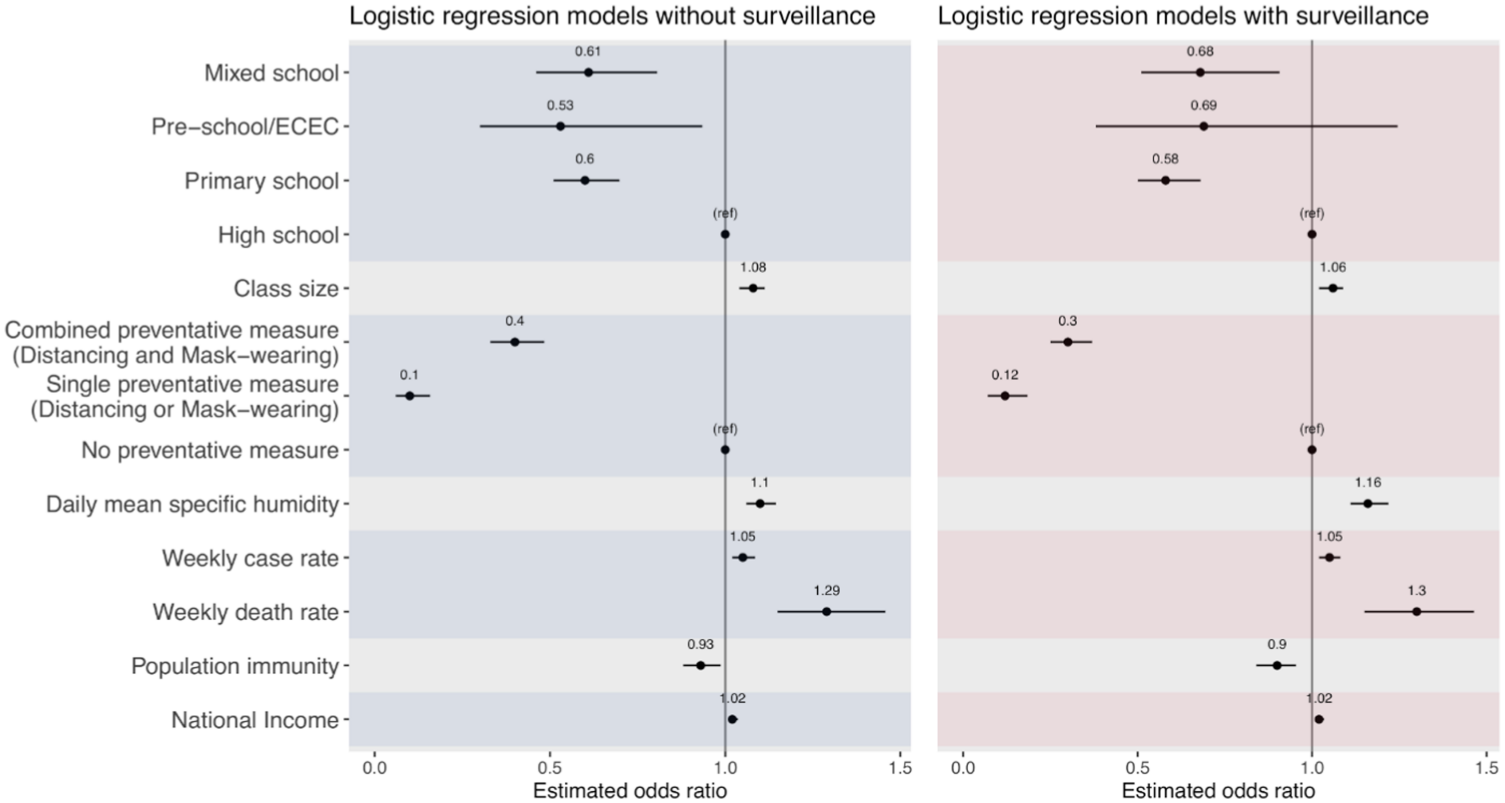
Odds ratio estimates from the marginal models. Left panel shows results from models without adjusting for surveillance, and right panel shows results from corresponding models additionally adjusting for surveillance. Black dots show the mean odds ratio estimates, and horizontal black bars show the 95% confidence intervals. The vertical black bar in each plot indicates the null value of 1.0. Each set of models is delineated by alternating the shaded regions

As noted above in the conceptual analysis, the type of surveillance policy implemented in schools could affect the reported SAR in both directions. Thus, we included surveillance type in all models. However, as a sensitivity analysis, we also tested each model without surveillance type included. Results for both versions are reported in Figure 3.

#### 2.3.2 Multirisk factor analysis

All seven variable groups described above were found to be associated with SAR in the marginal analysis (see Section 3). We thus tested models including different combinations of these variables to identify a multirisk model that best explains the observed SAR. For all models, we included surveillance type to account for potential biases in reporting including missing asymptomatic infections, which would underestimate SAR. We also assessed for confounding between our variables of interest and SARS‐CoV‐2 SAR (see adjustments specified above). This procedure tested all possible combinations of significant variables identified from the marginal analysis. We then evaluated and selected the most parsimonious model with the best fit based on the Akaike information criterion (AIC; Table S1). The best performing model took the following form:

l𝑜𝑔𝑖𝑡(𝑆𝐴𝑅)∼school type+preventative measures+surveillance+seasonal changes+weekly case rate+population immunity+individualism.

All statistical analyses were performed in RStudio, a user interface for R (R Foundation for Statistical Computing, Vienna, Austria). All models were fitted using the “glm” function from the built‐in “stats” library in R.

#### 2.3.3 Sensitivity analysis

We tested different measures of community transmission, to examine the robustness of our model results to potential biases due to variations in case‐ascertainment, mortality risk, and delay in event occurrence (e.g., from infection to death^40^) and reporting. Specifically, for the best‐performing multirisk factor model, we additionally examined three other measures in representing the intensity of community transmission, in lieu of weekly case rate during the extended time period (i.e., extending the end of study period by 2 weeks): (1) weekly death rate during the extended time period, (2) weekly death rate during the study period (i.e., without the 2‐week extension), and similarly, (3) weekly case rate during the study period.

## 3 RESULTS

### 3.1 Summary statistics

We identified 35 reported SARS‐CoV‐2 outbreaks in schools, totaling 728 secondary cases in children among 21,600 contacts. These outbreaks occurred in 12 countries, spanning four WHO regions including the Americas, Western Pacific, European, and Eastern Mediterranean Region. Figure 1 shows the study site, school type, study period, and reported SARS‐CoV‐2 SAR for each included outbreak. Table 1 shows the frequencies and summary statistics for SARS‐CoV‐2 SAR and other variables included. While the reported SAR ranged from 0% to 100%, the majority of schools reported very low SAR (median: 2%, interquartile range: 0–8%). Roughly even proportion of different school types were included: 5 (14.2%) were preschools, 10 (28.6%) were primary schools, 10 (28.6%) were high schools, and 10 (28.6%) were mixed schools. The majority of schools tested all contacts of the index cases (21/35 or 60%), and the majority required at least one preventive measure (26/35 or 74.3%).

**TABLE 1:** Characteristics of school outbreaks and related risk factors

Study characteristic	n (%), N = 35
Surveillance	
Symptomatic or some asymptomatic	14 (40%)
All contacts	21 (60%)
School type	
High school	10 (29%)
Primary school	10 (29%)
Preschool/ECEC	5 (14%)
Mixed school	10 (29%)
Preventative measure	
No preventative measure	9 (26%)
Single preventative measure (distancing or mask wearing)	12 (34%)
Combined preventative measure (distancing and mask wearing)	14 (40%)
Individualism (>77)	19 (54%)
	Median (interquartile range)
Secondary attack rate	0.02 (0.00, 0.08)
Population immunity rate (per 100 people)	0.51 (0.03, 1.96)
Weekly case rate (per 10,000 people)	3 (0, 14)
Weekly death rate (per 100,000 people)	0.24 (0.02, 1.62)
Daily mean specific humidity (g/kg)	6.94 (4.14, 9.97)
National income (thousand £)	41 (38, 53)
Average class size	20.0 (19.2, 23.4)

Abbreviation: ECEC, early childhood education center.

### 3.2 Marginal analysis

The marginal analysis with or without adjusting for surveillance generated similar estimates (Figure 3). Thus, below, we present results adjusting for surveillance. This analysis identified several associating factors that are directly related to schools, including school type, class size, preventive measures, and seasonal changes. For school type, compared with high schools, being in preschools (adjusted odds ratio [aOR]: 0.69, 95% CI: 0.38–1.25), primary schools (aOR: 0.58, 95% CI: 0.50–0.68), or mixed schools (aOR: 0.68, 95% CI: 0.51–0.91) was associated with a lower risk of SARS‐CoV‐2. For the school physical setting measure, each 1‐person increase in the national average class size was associated with an increased risk of contracting SARS‐CoV‐2 in schools (aOR: 1.06, 95% CI: 1.02–1.09). Single (distancing or masking) and combined preventive measure (distancing and masking) were both associated with a lower SAR in schools, with an aOR of 0.12 (95% CI: 0.07–0.18) and 0.30 (95% CI: 0.25–0.37), respectively. For disease seasonality, which could affect the transmission in schools and the community in general, each 1 g/kg increase in specific humidity was associated with an increased risk of contracting SARS‐CoV‐2 in schools (aOR: 1.16, 95% CI: 1.11–1.22); however, we note that specific humidity was low during most outbreaks included in this study (median: 6.94 and interquartile range [IQR]: 4.14–9.97 g/kg; see Table 1).

In addition, the marginal analysis also identified several associating factors, indirectly related to schools via the community/population. For the intensity of community transmission, both higher COVID‐19 case rate and death rate in the community were associated with an increased risk of contracting SARS‐CoV‐2 in schools (aOR = 1.05, 95% CI: 1.02–1.08, for cases per 10,000 people per week; and aOR = 1.30, 95% CI: 1.15–1.46, for deaths per 100,000 people per week). Individualism was included in four models based on the conceptual analysis (see Section 2 and Figure 2); all four models showed that higher level of individualism was associated with an increased risk (mean aOR ranged from 2.72 to 6.67, and all 95% CI had a lower bound >1). Higher national income (aOR: 1.02, 95% CI: 1.01–1.03 per 1000 British pounds) were associated with an increased risk; note, however, all outbreaks included here occurred in developed regions (Figure 1). Conversely, higher prior population immunity (using cumulative case rate per 100 people as a proxy, aOR: 0.90, 95% CI: 0.84–0.95) was associated with a decreased risk.

### 3.3 Multirisk factor analysis

Among all models tested (Table S1), the best‐performing model with the lowest AIC included six key groups of risk factors, namely, school type, preventive measures, seasonality, intensity of community transmission, population immunity, and individualism, adjusting for surveillance.

**FIGURE 4:**
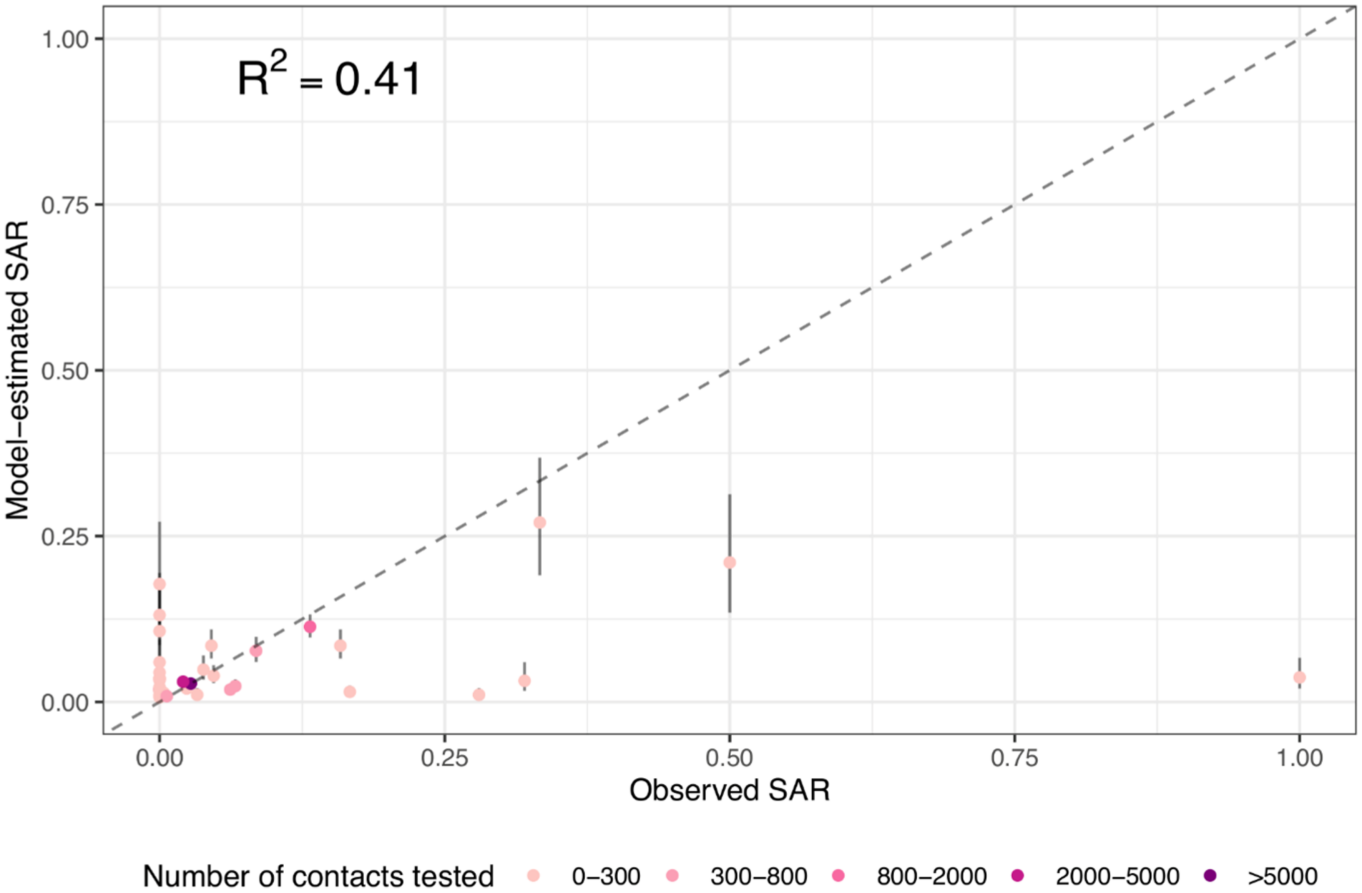
Model fit of the best‐performing multirisk model. Dots show the fitted SAR for each outbreak (*y*‐axis), compared with the observed SAR (*x*‐axis), and bars around each dot show the 95% confidence intervals of model estimates. The color of each dot indicates the number of contacts tested in each outbreak (see legend). The McFadden's pseudo‐*R* ^2^ is computed using eq. 30 in McFadden^41^

**FIGURE 5:**
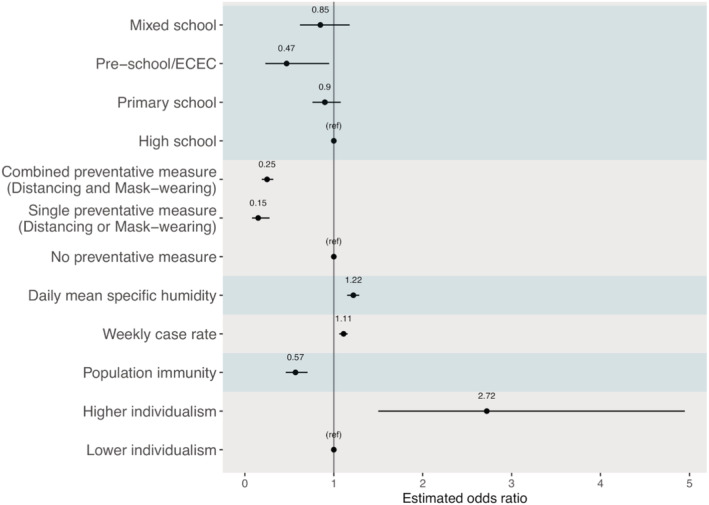
Odds ratio estimates from the best‐performing multi‐risk model. Black dots show the mean odds ratio estimates, and horizontal black bars show the 95% confidence intervals. The vertical black bar indicates the null value of 1.0. Each variable type is delineated by the shaded regions

Overall, these risk factors in combination were able to explain 41.0% of the variance in the reported SARS‐CoV‐2 SAR (McFadden's pseudo *R* ^2^ = 0.41; Figure 4).^41^, ^42^ The estimated aORs for each risk factor are shown in Table 2 and Figure 5. The sensitivity analysis shows consistent estimates across models using different measures of community transmission (see Table S2).

**TABLE 2:** Results of the best‐fit multirisk factor model for the identification of factors associated with SARS‐CoV‐2 SAR in schools

Variable	aOR (95% CI)
School type	
Mixed school	0.85 (0.62, 1.18)
Preschool/ECEC	0.47 (0.23, 0.95)
Primary school	0.9 (0.76, 1.08)
High school	Reference
Preventative measures	
Combined preventative measure (distancing and mask wearing)	0.25 (0.19, 0.32)
Single preventative measure (distancing or mask wearing)	0.15 (0.08, 0.28)
No preventative measure	Reference
Seasonal changes	
Daily mean specific humidity	1.22 (1.15, 1.29)
Community transmission	
Weekly case rate	1.11 (1.06, 1.16)
Population immunity	
Population immunity	0.57 (0.46, 0.71)
Individualism	
Higher individualism	2.72 (1.5, 4.95)
Lower individualism	Reference
Surveillance	
All contacts	3.02 (2.13, 4.28)
Symptomatic or some asymptomatic	Reference

*Note*: Adjusted odds ratio (aOR) estimates and 95% confidence intervals are given from the logistic regression model including surveillance to control for differences in testing school clusters, school type due to inconsistent reporting of age groups in the literature, number of preventative measures implemented in schools, and characteristics of the study sites (i.e., level of individualism, daily mean specific humidity, population immunity, and weekly case rates per 10,000).

Abbreviation: ECEC, early childhood education center.

Consistent with the marginal analysis, the best‐fit multirisk factor model showed that higher COVID‐19 case rate in the community (aOR: 1.11, 95% CI: 1.06–1.16; for 1 additional case reported among 10,000 people each week) and higher level of individualism (aOR: 2.72, 95% CI: 1.50–4.95; above vs. below the mean) were associated with an increased risk of SARS‐CoV‐2 infection in schools. Conversely, both single (aOR: 0.15, 95% CI: 0.08–0.28) and combined (aOR: 0.25, 95% CI: 0.19–0.32) preventive measures were associated with a reduced risk of SARS‐CoV‐2 infection in schools. In addition, higher population immunity (aOR: 0.57, 95% CI: 0.46–0.71, using cumulative case rate per 100 people as a proxy) was also associated with a reduced risk of SARS‐CoV‐2 infection in schools. Compared with students in high schools, the aOR was 0.47 (95% CI: 0.23–0.95) for students in preschools, 0.90 (95% CI: 0.76–1.08) for students in primary schools, and 0.85 (95% CI: 0.62–1.18) for students in mixed schools. As expected, reported SARs were higher when all contacts were tested regardless of symptoms (aOR: 3.02, 95%: 2.13–4.28; vs. only testing symptomatic contacts or only a portion of asymptomatic contacts).

## 4 DISCUSSION

Leveraging available data on multiple reported SARS‐CoV‐2 outbreaks in schools and potential risk factors, we have examined main factors associated with the risk of SARS‐CoV‐2 transmission in schools. Our analyses suggest that SARS‐CoV‐2 SAR in schools was associated with both preventative measures in schools and population factors including the level of community transmission, individualism, and population immunity, once adjusted for surveillance and school type.

Foremost, we identified several population or community factors to be highly associated with SARS‐CoV‐2 transmission in schools, all of which point to the importance of communal efforts to collectively reduce the risk of transmission and protect children in schools. In particular, all models (in both the marginal analysis and the multirisk factor analysis) consistently showed that higher level of individualism of the population was associated with higher SARs in schools. This finding is consistent with a recent study linking collectivism (vs. individualism) to usage of preventive measures like mask use during the COVID‐19 pandemic.^43^ Along similar lines, the models associated higher transmission in the community with higher SARs in schools, suggesting the potential community‐to‐school importation of cases and subsequent risk of outbreak in schools. As such, care must be applied when reopening or operating schools in areas with high levels of community transmission. In addition, reversing some original fears about school‐to‐community SARS‐CoV‐2 transmission, it is likely that the community transmission drives outbreaks in school, not the reverse. Further, the models showed that higher population immunity, which could lower transmission overall, was associated with lower SARs in schools. With the availability of COVID‐19 vaccines, predominantly to adults and older children at present, it is paramount that all eligible adults get vaccinated promptly to lower the risk of transmission in the community and, in turn, to provide indirect protection to children via the increased population immunity.

Our models estimated a substantial transmission reduction in schools when both distancing and mask wearing were required (aOR: 0.25, 95% CI: 0.19–0.32) or when distancing alone was required (note the aOR for either measure alone was 0.15 [95% CI: 0.08–0.28], with the majority of schools in this category [10 of 12] requiring distancing alone). Thus, both estimates indicate the importance of distancing. This finding is likely a combined outcome of reduced number of contacts and reduced short‐range transmission when social distancing policies were followed. Maintaining distance has often necessitated fewer people in a room at the same time and/or reduced time spent in schools (e.g., when rotation‐based schedules are implemented), leading to fewer contacts. In addition, the increased personal space in classroom enables students to avoid the likely higher viral concentration within short‐range of the emitter (either via aerosols, droplets, or in combination) when far apart. Nevertheless, it is important to note that social distancing measures may be more difficult to achieve fully in disadvantaged communities (often of color) with underfunded and overcrowded schools.^44^, ^45^ Furthermore, racially motivated structural factors prevent these disadvantaged communities from practicing social distancing policies outside of the school. For example, these communities tend to make up most of essential workers and thus have higher rates of transmission in their community,^46^ increasing potential introduction of infections into schools.

In comparison with high schools, students in both preschools and primary schools had a lower risk of SARS‐CoV‐2 infection (Figures 3 and 5). This finding is consistent with previous studies indicating the likely lower susceptibility to SARS‐CoV‐2 infection and transmissibility among young children.^4^, ^5^ In addition, it is also likely in part due to the greater ability of older children to follow directions regarding preventive measures but with less compliance among high school students (e.g., after accounting for factors including preventive measures, the estimated risk differences across school types were less pronounced; see Figure 5 vs. Figure 3).

This study has several limitations. First, all school outbreaks included in this analysis (n = 35) occurred prior to the emergence and widespread circulation of the more transmissible SARS‐CoV‐2 variants of concern (e.g., the delta and omicron variants). We are thus unable to estimate variant‐specific impacts. Nonetheless, even though the magnitude of impact may alter somewhat due to changes in circulating SARS‐CoV‐2 variants, the identified risk factors and their relative importance to school transmission likely would still hold given the robust risk mechanisms. Second, we were unable to estimate the impact of distancing and mask wearing separately, due to the small sample size of schools that required masking alone (n = 2). Third, due to a lack of detailed information for each specific school setting, we used proxy measures in the analyses (e.g., class size at the national level was used rather than for each reporting school), which may have limited the ability of the models to identify the association of these factors with SARS‐CoV‐2 transmission risk. Similarly, due to the lack of data, we were not able to examine other key factors such as ventilation in classrooms, social economic status of individual students and their households, and potential differences in susceptibility and transmissibility by age group. Future work with comprehensive study designs and data collection is warranted to provide further insights into how infections, not limited to SARS‐CoV‐2, spread in schools and the broad, bidirectional impact of school and community transmission. This would be invariable to inform better strategies to combat future infectious disease outbreaks.

## AUTHOR CONTRIBUTIONS

**Haokun Yuan:** Formal analysis; investigation; methodology; validation; visualization. **Connor Reynolds:** Formal analysis; investigation; methodology; validation; visualization. **Syndey Ng:** Formal analysis; investigation; methodology; validation; visualization. **Wan Yang:** Conceptualization; funding acquisition; investigation; methodology; supervision; validation.

### PEER REVIEW

The peer review history for this article is available at https://publons.com/publon/10.1111/irv.12968.

## Supporting information


**Table S1.** Performance of different models. In the marginal analysis, 8 models for 7 groups of risk factors were tested (note weekly case rate and weekly death rate, both representing community transmission, were tested separately in two models). In the multi‐risk factor analysis, 19 models with all possible combinations of 6 major groups of risk factors tested significant (i.e., when a risk group was a subset of a larger one, only the larger risk group was tested). All models adjusted for surveillance. The best‐performing model with the lowest AIC is bolded.
**Table S2.** Sensitivity analysis for both cases and deaths, with and without a 2‐week extension to the study time period.

**Data S1**. **Supplementary data.** Data for all school outbreaks included in this study and related risk factors.

## Data Availability

All data are included in the supplementary dataset.
