# Supplementary material for: Factors affecting the transmission of SARS‐CoV‐2 in school settings
Source: Influenza Other Respir Viruses. 2022 Feb 10;16(4):643–52. doi: 10.1111/irv.12968 (PMC9111692; doi:10.1111/irv.12968)
Supplement: Supplementary file 1 — Table S1. Performance of different models. In the marginal analysis, 8 models for 7 groups of risk factors were tested (note weekly case rate and weekly death rate, both representing community transmission, were tested separately in two models). In the multi‐risk factor analysis, 19 models with all possible combinations of 6 major groups of risk factors tested significant (i.e., when a risk group was a subset of a larger one, only the larger risk group was tested). All models adjusted for surveillance. The best‐performing model with the lowest AIC is bolded. Table S2. Sensitivity analysis for both cases and deaths, with and without a 2‐week extension to the study time period. [file IRV-16-643-s001.docx]

# **Supplemental Tables**

# **for**

**Factors affecting the transmission of SARS-CoV-2 in school settings**

**Table S1.** Performance of different models. In the marginal analysis, 8 models for 7 groups of risk factors were tested (note weekly case rate and weekly death rate, both representing community transmission, were tested separately in two models). In the multi-risk factor analysis, 19 models with all possible combinations of 6 major groups of risk factors tested significant (i.e., when a risk group was a subset of a larger one, only the larger risk group was tested). All models adjusted for surveillance. The best-performing model with the lowest AIC is bolded.

| **Analysis** | **Model (all adjusted for surveillance)** | **AIC** |
| --- | --- | --- |
| Marginal analysis | Preventative measures, Daily mean specific humidity, Individualism | 494.0 |
|  | Weekly case rate, Individualism, Population immunity | 705.4 |
|  | Weekly death rate, Individualism, Population immunity | 699.6 |
|  | Daily mean specific humidity | 689.7 |
|  | Class size, National income, Daily mean specific humidity, Individualism | 583.0 |
|  | School type | 687.5 |
|  | National income | 718.9 |
|  | Population immunity, Individualism | 713.0 |
| Multi-risk factor analysis | Weekly case rate, Individualism, Population immunity, National income | 691.5 |
|  | Daily mean specific humidity, National income | 671.5 |
|  | Weekly case rate, Individualism, Population immunity, School type | 667.9 |
|  | School type, National income | 665.5 |
|  | Daily mean specific humidity, School type | 660.9 |
|  | Daily mean specific humidity, Weekly case rate, Individualism, Population immunity | 644.0 |
|  | Weekly case rate, Individualism, Population immunity, School type, National income | 642.4 |
|  | Daily mean specific humidity, Weekly case rate, Individualism, Population immunity, National income | 634.6 |
|  | Daily mean specific humidity, School type, National income | 623.3 |
|  | Daily mean specific humidity, Weekly case rate, Individualism, Population immunity, School type | 609.5 |
|  | Daily mean specific humidity, Weekly case rate, Individualism, Population immunity, School type, National income | 590.4 |
|  | Preventative measures, Daily mean specific humidity, Individualism | 494.0 |
|  | Preventative measures, Daily mean specific humidity, Individualism, National income | 493.8 |
|  | Preventative measures, Daily mean specific humidity, Individualism, School type, National income | 488.0 |
|  | Preventative measures, Daily mean specific humidity, Individualism, School type | 486.0 |
|  | Preventative measures, Daily mean specific humidity, Individualism, Weekly case rate, Population immunity, National income | 454.5 |
|  | Preventative measures, Daily mean specific humidity, Individualism, Weekly case rate, Population immunity, School type, National income | 454.3 |
|  | Preventative measures, Daily mean specific humidity, Individualism, Weekly case rate, Population immunity | 452.7 |
|  | **Preventative measures, Daily mean specific humidity, Individualism, Weekly case rate, Population immunity, School type** | **452.6** |

**Table S2.** Sensitivity analysis for both cases and deaths, with and without a 2-week extension to the study time period.

|  | **Variable** | **Without extension** | | **With extension** | |
| --- | --- | --- | --- | --- | --- |
|  |  | **aOR (95%CI)** | **AIC** | **aOR (95%CI)** | **AIC** |
| case | Weekly case rate | 1.04 (0.99, 1.08) | 475.2 | 1.11 (1.06, 1.16) | **452.6** |
|  | Single preventative measure (Distancing or Mask-wearing) | 0.09 (0.04, 0.18) |  | 0.15 (0.08, 0.28) |  |
|  | Combined preventative measure (Distancing and Mask-wearing) | 0.32 (0.24, 0.41) |  | 0.25 (0.19, 0.32) |  |
|  | All contacts | 2.31 (1.67, 3.19) |  | 3.02 (2.13, 4.28) |  |
|  | Primary school | 0.89 (0.75, 1.06) |  | 0.9 (0.76, 1.08) |  |
|  | Pre-school/ECEC | 0.38 (0.19, 0.76) |  | 0.47 (0.23, 0.95) |  |
|  | Mixed school | 0.69 (0.5, 0.95) |  | 0.85 (0.62, 1.18) |  |
|  | Population immunity | 0.74 (0.6, 0.9) |  | 0.57 (0.46, 0.71) |  |
|  | Daily mean specific humidity | 1.21 (1.15, 1.28) |  | 1.22 (1.15, 1.29) |  |
|  | Higher individualism (>77) | 5.4 (2.78, 10.48) |  | 2.72 (1.5, 4.95) |  |
| death | Weekly death rate | 1.11 (0.91, 1.35) | 476.8 | 1.21 (1.04, 1.41) | 472.6 |
|  | Single preventative measure (Distancing or Mask-wearing) | 0.07 (0.04, 0.14) |  | 0.08 (0.04, 0.16) |  |
|  | Combined preventative measure (Distancing and Mask-wearing) | 0.35 (0.28, 0.44) |  | 0.34 (0.27, 0.43) |  |
|  | All contacts | 2.25 (1.64, 3.08) |  | 2.32 (1.69, 3.19) |  |
|  | Primary school | 0.9 (0.75, 1.08) |  | 0.91 (0.76, 1.09) |  |
|  | Pre-school/ECEC | 0.39 (0.19, 0.77) |  | 0.4 (0.2, 0.8) |  |
|  | Mixed school | 0.62 (0.46, 0.85) |  | 0.61 (0.45, 0.83) |  |
|  | Population immunity | 0.83 (0.75, 0.92) |  | 0.82 (0.74, 0.91) |  |
|  | Daily mean specific humidity | 1.2 (1.14, 1.27) |  | 1.2 (1.14, 1.27) |  |
|  | Higher individualism (>77) | 6.67 (3.75, 11.86) |  | 5.5 (3.12, 9.69) |  |
